# Effects of Occlusal Plane Inclination on the Temporomandibular Joint Stress Distribution: A Three-Dimensional Finite Element Analysis

**DOI:** 10.1155/2022/2171049

**Published:** 2022-09-02

**Authors:** Ebru Demet Cifter

**Affiliations:** Istanbul University Faculty of Dentistry, Department of Prosthodontics, Istanbul, Turkey

## Abstract

**Background:**

Sudden changes in masticatory loads and occlusal conditions contribute to temporomandibular disorders. Clockwise (CW) or counterclockwise (CCW) rotation of the occlusal plane is one of the factors that alter the direction of the occlusal forces transmitted to the temporomandibular joint structures. Finite element analysis was used to identify possible regions of high stress in the temporomandibular joint.

**Materials and Methods:**

A computer-aided design model of a symmetrical edentulous maxillomandibular bony complex with a temporomandibular joint was manually generated using Rhinoceros 4.0 freeform modeling software. Three-dimensional discrete mesh generation was performed using VRMesh Studio. The reference occlusal plane angle was accepted as 8° in the sagittal plane, and by modifying 4° in the CW and CCW directions, CW and CCW models were obtained, respectively. The present study aimed to evaluate the changes in stress distribution in the condylar cartilage and temporomandibular disc using the von Mises and maximum-minimum principal stress evaluations of three different occlusal plane inclinations. The null hypothesis of this three-dimensional finite element analysis was that “occlusal plane inclination does not change the stress distribution on the temporomandibular joint structures.”

**Results:**

The compressive stress on the condyle increased with CW rotation of the occlusal plane. The von Mises equivalent stress of the temporomandibular disc shifted to the medial, posterior, and superior directions after CW and CCW rotations of the occlusal plane. The CW rotation of the occlusal plane increased the von Mises equivalent.

## 1. Introduction

The temporomandibular joint (TMJ) is one of the most complex joint systems in the body, connecting a single bone to the skull with two separate joints. It performs gliding and hinging movements; therefore, it is called a ginglymoarthrodial joint, which provides special biomechanical features. There is a significant relationship between the dentition alignment and the distribution of masticatory forces through the stomatognathic system. The harmony of condylar guidance with anterior guidance and occlusal plane (OP) inclination maintains the structural health of the system. Sudden changes in masticatory loads and occlusal conditions are listed as contributing factors to temporomandibular disorders [1].

OP is defined by The Glossary of Prosthodontic Terms as follows: “The average plane established by the incisal and occlusal surfaces of the teeth” [2]. The OP is at an angle of approximately 8 ± 4° relative to the Frankfort horizontal plane when viewed in the sagittal plane [3]. The angle of the plane can be altered by full-arch prosthodontic rehabilitation, tooth loss, orthodontic treatment, or during orthognathic surgeries, correcting low or high occlusal facial types [4]. The clockwise (CW) or counterclockwise (CCW) rotation of the OP inclination changes the direction of occlusal forces transferred to the TMJ additional to noticeable esthetic outcomes [5, 6]. Changes in the direction of the occlusal forces and the decrease of OP angle are assumed to be related to increased pressure in TMJ structures [7]. Barientos et al. stated that the direction of the clenching forces affects the localization of stress distribution areas on TMJ structures [8]. In the TMJ, the disc and articular cartilage are the load-bearing structures. They are responsible for stress absorption and dissipation of the energy produced during function [9]. Excessive loads, orthopedic instability, and aging are the major reasons for the degeneration of the TMJ components. Erosion and roughening of the articular structures can lead to pain and dysfunction, which can later be accompanied by inflammatory changes [10].

Finite element analysis (FEA) is an effective, noninvasive, and qualitative method used for identifying the possible region of high stresses that are responsible for soft tissue and bone degeneration in a complex structure such as the TMJ [11]. To evaluate the possible relationship between OP inclination and TMJ biomechanics, a three-dimensional (3D) finite element model was constructed. The present study aimed to evaluate the changes in the stress distribution in condylar cartilage and temporomandibular disc by the von Mises and maximum-minimum principal stress (MaxPs-MinPs) evaluations of three different OP inclinations. The null hypothesis of this 3D FEM analysis was that “occlusal plane inclination does not change the stress distribution on the TMJ structures.”

## 2. Materials and Methods

A computer-aided design model of a symmetrical edentulous maxillomandibular bony complex with the TMJ was manually generated using Rhinoceros 4.0 freeform modeling software (3670 Woodland Park Ave N, Seattle, WA 98103, USA). The anatomical data used for this process were gathered from sagittal and coronal tracings and images of the Sobotta Anatomy Atlas and the Visible Human Project (US National Library of Medicine, Bethesda, MD, USA). Cortical and trabecular bone layers were determined using Rhinoceros software. Cortical bone thickness was defined as 2 mm for the glenoid fossa, condylar head, and mandible. A 0.2 mm uniform layer of articular cartilage was created at the glenoid fossa and condyle to maximize the realism of the created model based on the anatomic findings [12, 13]. The temporomandibular disc, retrodiscal tissue, and capsular ligament were modeled manually between the glenoid fossa and condyle using the same software in accordance with the surfaces of the glenoid fossa and condyle which were modeled from the Sobotta Anatomy Atlas and the Visible Human Project (US National Library of Medicine, Bethesda, MD, USA).

**Figure 1:**
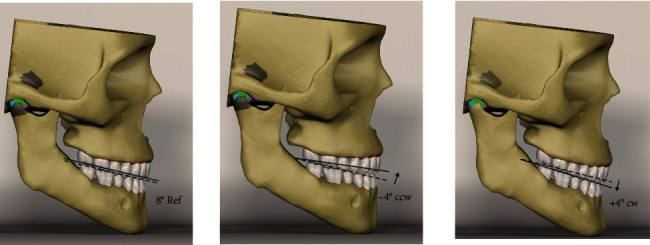
Reference model (8° ref), counterclockwise (−4° CCW), and clockwise (+4° CW) models.

Modeling of the dentition was manually accomplished, as suggested by the Rhinoceros software. The 3D model parts were created in STL (stereolithography) format, exported to Rhinoceros software, and integrated using the Boolean method. While integrating the denture into the alveolar bone, a periodontal membrane was formed with a thickness of 0.25 mm and was assumed to be even in all regions [14]. Each tooth was positioned in the maxilla and mandible corresponding to the alveolar arch shape in accordance with the Roth prescription (Table 1) [15]. While integrating the denture into the alveolar process, the interocclusal relationship was set according to the Angle Class I molar relationship. The reference OP angle was accepted as 8° in the sagittal plane, and by changing 4° in the CW and CCW directions, CW and CCW models were obtained, respectively (Figure 1).

After completing the modeling process, 3D discrete mesh generation was performed using the VRMesh Studio (VirtualGrid Inc., Bellevue City, WA, USA) software. In the regions of interest, 8-node brick type hexahedral elements were used as much as possible. Fewer knotted elements were used in the regions close to the center of the structure. This modeling technique aims to create a high-quality network structure with the highest possible number of nodes to facilitate calculations. The total number of elements and nodes used in the mathematical model is listed in Table 2.

After the mesh convergence analysis process, the model was exported to Algor Fempro software (Algor Inc., USA) in STL format to perform the FEA. Before the FEA, the material properties were assigned to all structures composing the 3D model. The elastic moduli and Poisson's ratios of the elements are listed in Table 3.

All the materials in this study were assumed to be homogeneous, isotropic, and linearly elastic. To limit the displacement of the model in the loading simulations, boundary conditions were assigned to the nodes on the posterior border of the temporal bone and the most superior part of the model as a zero displacement in all directions. The model was designed symmetrically along the *x* axis. The applied masticatory muscle loads are presented in Table 4.

## 3. Results

Stress distribution on the condylar cartilage and TMJ disc was evaluated by maximum and minimum principal stress (MaxPs and MinPs, respectively) and the von Mises stress calculations. The highest and lowest amounts of maximum and minimum principal stress and von Mises stress under the three different occlusal inclinations are presented in Table 5.

### 3.1. Condylar Cartilage

The MaxPs values indicating tensile stresses in the mandibular structure were localized on the frontal face of the condylar cartilage in all models. CCW and CW rotation of the OP did not change the quantity or location of the highest MaxPs values.

The CW rotation of the OP increased the lowest value of MaxPs more than the CCW rotation without changing the location where it remained on the posterior face of the condylar neck.

**Figure 2:**
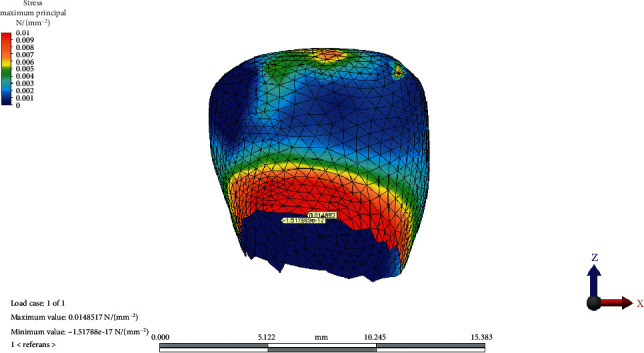
Maximum and minimum values and maximum principal stress distribution on condylar cartilage in the reference model.

**Figure 3:**
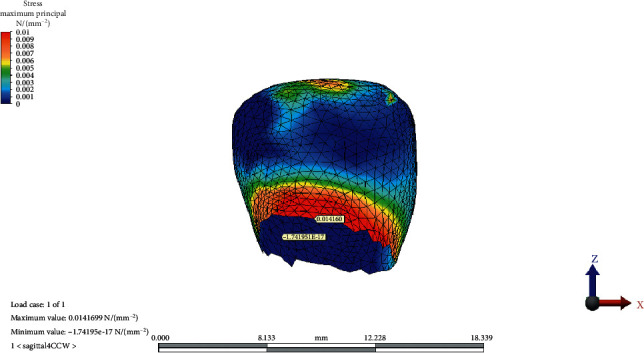
Maximum principal stress distribution on condylar cartilage after counterclockwise rotation of the occlusal plane.

**Figure 4:**
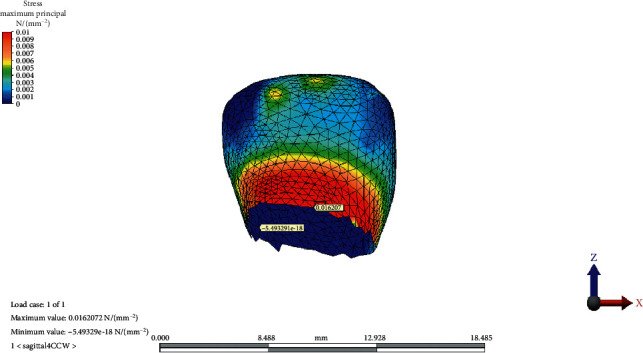
Maximum principal stress distribution on condylar cartilage after clockwise rotation of the occlusal plane.

The MaxPs distribution on the condylar cartilage in the reference model, CCW rotation, and CW rotation of the occlusal plane is presented in Figures 2–4, respectively.

**Figure 5:**
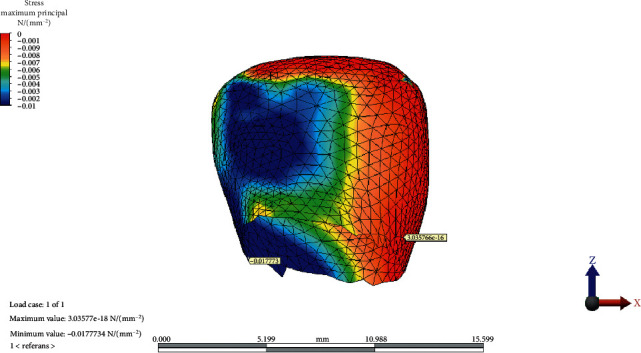
Minimum principal stress distribution on condylar cartilage in the reference model.

**Figure 6:**
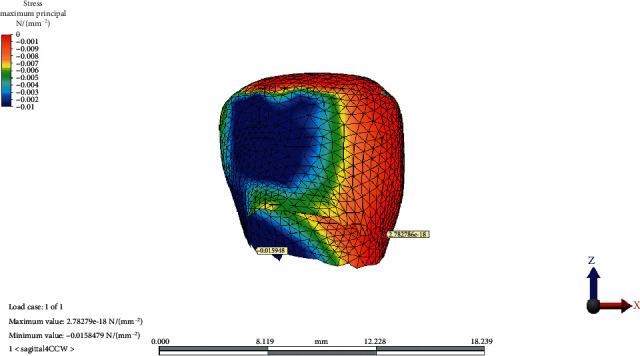
Minimum principal stress distribution on condylar cartilage after counterclockwise rotation of the occlusal plane.

**Figure 7:**
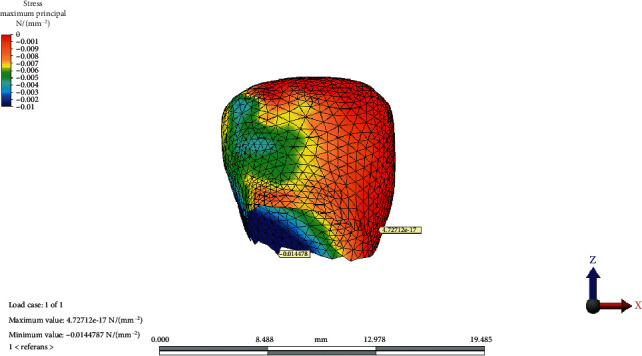
Minimum principal stress distribution on condylar cartilage after clockwise rotation of the occlusal plane.

The distribution of MinPs values in the condylar cartilage in the reference model, CCW rotation, and CW rotation of the OP is presented in Figures 5–7, respectively.

The maximum values of the MinPs indicating the compressive stresses in the structure were the highest on the medial face of the condylar cartilage for all models and increased after CW rotation of the OP. The minimum values of the MinPs were on the lateral face of the condylar neck and did not change quantitatively following changes in the OP inclination.

### 3.2. Temporomandibular Joint Disc

The von Mises equivalent stresses were the highest on the lateral/inferior/frontal site of the TMJ disc in the reference model. The maximum stress point shifted medially, posteriorly, and superiorly as the OP inclined both CW and CCW. The greatest value for the maximum von Mises stress increased as the OP rotated CW. The minimum von Mises stress value was observed at the lateral-inferior/posterior site of the disc. The point moved medially, superiorly, and frontally as the OP inclined CCW; however, it did not change in place with the CW rotation of the OP.

**Figure 8:**
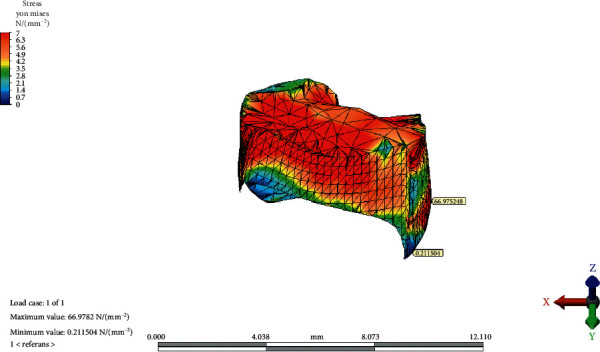
The maximum and minimum values and distribution of the von Mises stress on temporomandibular joint disc for the reference model.

**Figure 9:**
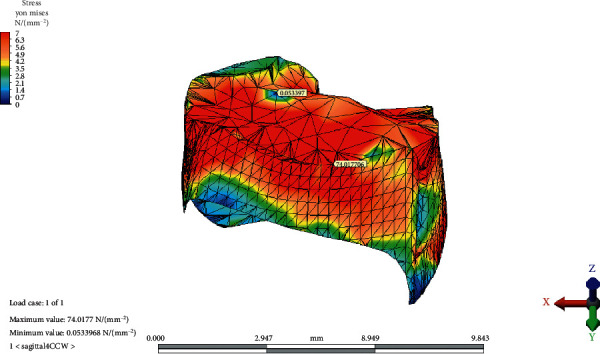
The maximum and minimum values and distribution of the von Mises stress on temporomandibular joint disc after counterclockwise rotation of the occlusal plane.

**Figure 10:**
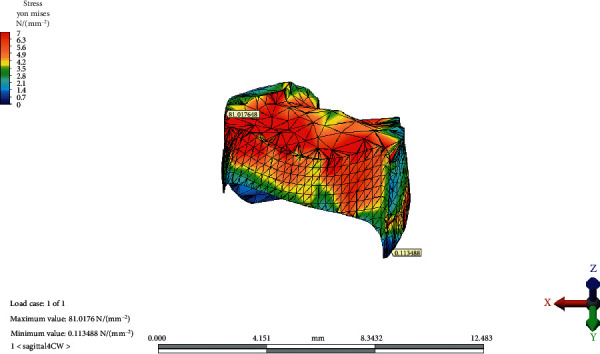
The maximum and minimum values and distribution of the von Mises stress on temporomandibular joint disc after clockwise rotation of the occlusal plane.

The maximum and minimum values and distribution of the von Mises stress on the TMJ disc for the reference model, CCW rotation, and CW rotation of the OP are presented in Figures 8–10, respectively.

## 4. Discussion

Computed simulations such as FEA have been successfully used to reveal the internal stresses of body structures for a long while [16]. Mathematical models provide the advantage of conducting studies that would never be possible to conduct on human subjects. Testing the same hypothesis by changing the variables under standardized conditions ensured the validity of the results.

The TMJ is one of the most complex joint systems in the human body. The internal structure of the joint can be monitored to a limited extent using imaging methods, such as magnetic resonance or computed tomography. The data obtained by imaging methods provide information about the instant status of the articular surfaces; however, they are unable to demonstrate the effects of changing forces. Furthermore, the FEA of the TMJ is limited by several assumptions. Adaptive changes in biological systems explain the reason for the variation in the occurrence of TMJ disorders among people with occlusal disharmonies. Nevertheless, the relationship between TMJ disorders and occlusal factors is still being comprehensively investigated [17].

The results of the current study revealed the change in the magnitude and direction of the occlusal forces transmitted to the TMJ disc and condylar cartilage is due to OP rotation. The tensile stresses on the condylar cartilage (MaxPs highest values) and the least compressive forces (MinPs lowest values) remained the same in magnitude and direction, suggesting that the condylar cartilage faces mostly compressive stresses in all models.

OP inclination changed the differential pattern of force applied on the TMJ disc both in direction and magnitude. CW rotation of the OP caused an increase in the maximum value of the MinPs, indicating compressive stress in the structure. CCW rotation slightly decreased the compressive stress on the condylar cartilage. Compressive stresses remained on the medial face of the condylar cartilage, regardless of the OP inclination.

CW rotation of the OP increased the von Mises stresses observed in the disc more than CCW rotation. The von Mises equivalent stresses were the highest on the lateral/inferior/frontal site of the TMJ disc in the reference model. This result is consistent with the findings of Mori et al. [9]. Maximum stress points shifted medially, posteriorly, and superiorly as the OP inclined both CW and CCW. The results of the current study are in line with the results of several studies that revealed the localization of stress patterns on the lateral side of the disc. In addition, occlusal trauma-induced thinning and perforation of the TMJ disc are found in the central and lateral areas in anatomical studies [18]. Furthermore, Hansson et al. found arthritic changes located mainly on the disc and in the lateral one-third of the joint from the cadaver studies [19]. The correlation between studies shows us that the disc is the most load-bearing element in the TMJ. Mori et al. conducted a FEA of cartilaginous tissues in the TMJ and suggested that the TMJ disc plays an important role in stress distribution during prolonged clenching [9]. According to the result of the current study, the increasing magnitude of the von Mises stress on the articular disc indicates that the major stress distributing element of the TMJ is the articular disc.

The change in the OP inclination in both directions (CCW and CW) shifted the maximum stress point of the disc in the opposite direction with increasing magnitudes. Nickel et al. stated that mediolateral translation due to tractional forces in the joint could affect degenerative changes in the cartilage structures [20]. Buranastidporn et al. concluded that arthritic changes of the articular disc were related to the compressive stress patterns of the clenching loads. Recurrent loading of the TMJ structures with incompatible forces is also suggested as the reason for internal derangement and disc displacement [21].

The significant energy dissipation capacity and stress relaxation observed under sheer and tensile loads protect the TMJ disc from irreversible structural deformation [21]. The adaptation capacity of the individual also plays an important role in the probability of developing temporomandibular disorders [1]. Patients with temporomandibular disorders should be evaluated closely during dental treatments which cause OP alteration. Unnecessary changes in the OP angle should be avoided during full-arch restorations in individuals with a healthy stomatognathic system. Although the etiology of temporomandibular disorders has not yet been established, increased joint loads are reported to be a possible reason for some temporomandibular disorders [22]. However, the role of occlusion-related changes in temporomandibular disorders remains controversial. To date, there is no certain scientific evidence that can specifically judge the relation between force distribution and temporomandibular disorders.

## 5. Conclusion

1. CW or CCW rotation of the occlusal plane did not change the tensile stresses on the condyle.
2. Compressive stresses on the condyle increased with the CW rotation of the OP; however, they remained on the medial side.
3. The von Mises equivalent stress of the TMJ disc shifted to medial, posterior, and superior directions after CW and CCW rotation of the OP.
4. In comparison to CCW, the CW rotation of the OP resulted in a greater increase of the von Mises equivalent stress.

## Figures and Tables

**Table 1:** Roth prescriptions of maxillary and mandibular teeth.

Roth prescriptions	Torque	Angulation
Maxillary centrals	+12°	5°
Maxillary laterals	+8°	9°
Maxillary cuspids	−2°	9°
Maxillary 1^st^/2^nd^ bicuspids	−7°	0°
Maxillary 1^st^/2^nd^ molars	−14°	0°
Mandibular incisors	0°	0°
Mandibular cuspids	−11°	7°
Mandibular 1^st^ bicuspids	−17°	0°
Mandibular 2^nd^ bicuspids	−22°	0°
Mandibular 1^st^ molars	−30°	1°
Mandibular 2^nd^ molars	−30°	0°

**Table 2:** The number of nodes and elements of clockwise (CW) and counterclockwise (CCW) models.

	Reference	CW	CCW
Number of nodes	231678	520627	472516
Number of elements	1066340	2104547	2061979

**Table 3:** The elastic moduli and Poisson ratios of the elements.

	Elastic modulus (MPa)	Poisson ratio
Cortical bone	13700	0.3
Enamel	84100	0.33
Periodontal ligament	0.0689	0.45
Cancellous bone	1370	0.3
Cartilage	7.4	0.4
TMJ disc	30.9	0.4
Retrodiscal tissues	1.5	0.4

**Table 4:** Masticatory muscle loads applied.

	Force applied ($\hat{N}$2)
Superficial masseter	190.40
Deep masseter	81.60
Medial pterygoid	174.80
Anterior temporalis	158
Middle temporalis	95.60
Posterior temporalis	75.60
Superficial masseter	190.40

**Table 5:** The highest and lowest amounts of maximum and minimum principal stress values on the condylar cartilage and von Mises stress values for temporomandibular joint (TMJ) disc (N/mm^2^).

	Reference	CCW^a^	CW^b^
MaxPs^c^ highest	0.01	0.01	0.01
MaxPs^c^ lowest	−1,51	−1,74	−5,49
MinPs^d^ highest	3,03	2,78	4,72
MinPs^d^ lowest	−0.01	−0.01	−0.01
vonM^e^ highest	66.97	74,01	81.01
vonM^e^ lowest	0.21	0.05	0.11

a: counterclockwise, b: clockwise, c: maximum principal stress d: minimum principal stress, e: von Mises stress.

## Data Availability

All data used to support the findings of the study will be made available upon reasonable request.
